# Bayesian-Optimized Hybrid Kernel SVM for Rolling Bearing Fault Diagnosis

**DOI:** 10.3390/s23115137

**Published:** 2023-05-28

**Authors:** Xinmin Song, Weihua Wei, Junbo Zhou, Guojun Ji, Ghulam Hussain, Maohua Xiao, Guosheng Geng

**Affiliations:** 1 College of Engineering, Nanjing Agricultural University, Nanjing 210031, China; 9203012015@stu.njau.edu.cn (X.S.); 2020112009@stu.njau.edu.cn (J.Z.); 2 College of Mechanical and Electronic Engineering, Nanjing Forestry University, Nanjing 210037, China; whwei@njfu.edu.cn; 3 Essen Agricultural Machinery Changzhou Co., Ltd., Changzhou 213000, China; jx9816@163.com; 4 Faculty of Mechanical Engineering, Ghulam Ishaq Khan Institute of Engineering Sciences & Technology, Topi 23460, Pakistan; gh_ghumman@hotmail.com

**Keywords:** Bayesian optimization, rolling bearing, fault diagnosis, hybrid kernel SVM

## Abstract

We propose a new fault diagnosis model for rolling bearings based on a hybrid kernel support vector machine (SVM) and Bayesian optimization (BO). The model uses discrete Fourier transform (DFT) to extract fifteen features from vibration signals in the time and frequency domains of four bearing failure forms, which addresses the issue of ambiguous fault identification caused by their nonlinearity and nonstationarity. The extracted feature vectors are then divided into training and test sets as SVM inputs for fault diagnosis. To optimize the SVM, we construct a hybrid kernel SVM using a polynomial kernel function and radial basis kernel function. BO is used to optimize the extreme values of the objective function and determine their weight coefficients. We create an objective function for the Gaussian regression process of BO using training and test data as inputs, respectively. The optimized parameters are used to rebuild the SVM, which is then trained for network classification prediction. We tested the proposed diagnostic model using the bearing dataset of the Case Western Reserve University. The verification results show that the fault diagnosis accuracy is improved from 85% to 100% compared with the direct input of vibration signal into the SVM, and the effect is significant. Compared with other diagnostic models, our Bayesian-optimized hybrid kernel SVM model has the highest accuracy. In laboratory verification, we took sixty sets of sample values for each of the four failure forms measured in the experiment, and the verification process was repeated. The experimental results showed that the accuracy of the Bayesian-optimized hybrid kernel SVM reached 100%, and the accuracy of five replicates reached 96.7%. These results demonstrate the feasibility and superiority of our proposed method for fault diagnosis in rolling bearings.

## 1. Introduction

With the development of the manufacturing industry, machine fault detection has become a very important field. Bearing, as a commonly used supporting part in machinery and equipment, has a great influence on the normal operation of the machine, while it also has a high incidence of failure because it often works under the condition of high speed and heavy load [1,2,3,4]. Statistics show that 30% of the failures of rotary machinery are related to bearings [5]. Therefore, fault diagnosis using vibration signals generated during its working process can reduce the probability of mechanical accidents and provide reliable decision support for later maintenance plans [6,7].

The most commonly used features for fault detection in rotating machines from vibration signals can be classified into three categories: time-domain, frequency-domain, and time-frequency domain features. Time-domain features include statistical features, such as mean, standard deviation, skewness, and kurtosis. Frequency-domain features include spectral features, such as power spectral density, frequency band energy, and frequency ratio. Time-frequency domain features include wavelet-based features, such as wavelet energy, wavelet entropy, and wavelet variance.

While these conventional features have been employed successfully in fault detection, there has been an increasing interest in the development of new methods for solving complex classification problems. One of these approaches is the Non-parallel Bounded Support Matrix Machine (NBSMM), which is a novel extension of SVMs that can effectively deal with non-linearly separable data by utilizing the concept of bounded support matrices. Another extension of SVMs is the multi-class fuzzy support matrix machine (MFSMM), which is a robust and efficient method for multi-class classification problems. The Convolutional-Vector Fusion Network (CVFN) is a recent development in the field of deep learning that combines the strengths of convolutional neural networks (CNNs) and vector fusion networks. CVFN is particularly effective in handling complex and heterogeneous data by fusing information from multiple modalities [8,9].

In addition to these new methods, kurtosis and Kullback–Liebler divergence have also been employed successfully in fault detection. Kurtosis is sensitive to the presence of impulsive signals, which are often associated with faults in rotating machines. High kurtosis values indicate the presence of impulsive signals, which can be used to detect faults, such as bearing faults and gear faults. Kullback–Liebler divergence has been used for fault detection in rotating machines by comparing the probability distribution functions of healthy and faulty signals. The Kullback–Liebler divergence between the two distributions can be used as a feature to detect faults [10,11].

On the other hand, with the continuous development of the Bayesian optimization (BO) algorithm, more and more researchers have begun to apply it to fault detection [12,13]. BO is a method used to optimize “black box” function which is defined as a function whose analytic expression is unknown. Therefore, we do not have access to their gradients. Hence, their evaluation, in terms of computing time and other resources, is costly. In addition, the evaluation of these functions may be subjected to noise pollution, which means that two evaluations at the same input location may yield varying results [14]. On the one hand, SVM is a kind of machine learning algorithm for classification and regression. BO can achieve the efficient optimization of “black box” functions by constructing a Gaussian process model to predict the value of unknown functions and selecting the next point for evaluation according to Bayes’ theorem. Additionally, SVM divides the data into two or more categories by finding the optimal decision hyperplane.

In relevant literature, we can see that many researchers have discussed the application of BO and SVM in the field of fault diagnosis. For example, Orhan et al. [15] employed BO and SVM algorithms for the diagnosis of motor faults. They used the BO algorithm to select the optimal SVM parameters and subsequently performed feature selection using the SVM algorithm. The SVM algorithm was then applied for motor fault diagnosis. Their results indicated that this approach exhibited high accuracy and robustness in diagnosing motor faults. Similarly, Li et al. [16] utilized BO and SVM algorithms for detecting rolling bearing faults. They applied the BO algorithm to optimize the SVM parameters and utilized statistics-based methods to extract vibration signal features from rolling bearings. Experimental results demonstrated that their approach effectively detected rolling bearing faults. Additionally, Xiong et al. [17] utilized the BO algorithm to improve the accuracy of bearing fault diagnosis by selecting the optimal SVM parameters. Their method automatically searched for the SVM parameters with the highest classification results. The approach yielded favorable results in bearing fault diagnosis. Furthermore, James Bergstra and Yoshua Bengio [18] proposed a technique based on random search and BO to optimize SVM hyperparameters and achieved excellent outcomes. Some researchers have also explored the combination of SVM with other machine learning techniques for fault diagnosis. For instance, Tian Han [19] proposed a method that combined improved SVM and convolutional neural networks for diagnosing rolling bearing faults and achieved favorable outcomes. Overall, relevant studies have demonstrated promising results when combining SVM with other machine learning techniques for fault diagnosis.

Although BO has advantages in fault diagnosis, it has some limitations. Firstly, the optimization of the Gaussian process in each iteration requires significant computing resources and time, particularly for large datasets. Secondly, BO is a global optimization algorithm based on probability and may fall into local optima, particularly for complex non-convex function problems. Thirdly, BO’s performance is highly reliant on parameter settings, such as the kernel function and hyperparameters of the Gaussian process. These settings can significantly impact the algorithm’s performance. Finally, BO requires prior knowledge to guide the search process, which can lead to decreased performance if the prior knowledge is insufficient or inaccurate. When using BO for fault diagnosis, it is essential to be aware of these limitations to better use the algorithm’s advantages and address the existing issues [20,21].

Our proposed theory focuses on the development of a Bayesian-optimized hybrid kernel SVM model with the aim to investigate its application in diagnosing faults in rolling bearings. To achieve this, we first decompose the vibration signal of rolling bearings into several time and frequency domain components using discrete Fourier transform (DFT). Then, permutation entropy obtained through decomposition is extracted as a feature vector. Next, we construct a hybrid kernel SVM model based on radial basis kernel function (RBF) and polynomial kernel function (Poly) kernels. We use the BO algorithm to optimize the penalty factor c, and the parameter coefficient g of the kernel function. Our fault diagnosis model for rolling bearings employs a hybrid kernel SVM approach, and we create the objective function using the Gaussian regression process of the BO algorithm. The objective function computes the mean square error of the verification set and utilizes the best network discovered during the optimization process and verification accuracy to determine the optimal penalty factor and core function parameters for the hybrid kernel SVM model. Following this, we train the hybrid kernel SVM model using the extracted feature vectors, and generate predictions for the test samples. We evaluate the feasibility of our proposed method through experiments that utilize the bearing data set from the Case Western Reserve University. We confirm the superiority of our proposed algorithm based on laboratory data and compare it with other fault diagnosis algorithms. Through these test cases, we comprehensively evaluate the feasibility and practicality of our proposed fault diagnosis method, providing valuable references for research and application in the field of bearing fault diagnosis. The strengths of our theory include the use of a hybrid kernel SVM approach with BO algorithm to optimize kernel function parameters, which has the potential to improve the accuracy of bearing fault diagnosis.

## 2. Theoretical Basis

### 2.1. Hybrid Kernel SVM

Support vector machine(SVM) is a binary classification model based on linear classifiers defined in feature space with maximum interval. If the following training data $\left\{x_{i},y_{i},i=1,2,\cdots ,n\right\}$ is given, where, $x_{i}\in R^{d}$ is the input value of the *i*th learning sample, and it is a d-dimensional column vector $x_{i}={\left[x_{i}^{1},x_{i}^{2},\cdots ,x_{i}^{d}\right]}^{T}$, and $y_{i}\in R$ is the corresponding target value. For nonlinear indivisible problems, x is mapped to a feature space by the nonlinear transformation Φ, thus transforming into a linear separable problem [22,23]. The linear estimation function can be defined as:

(1) $$y=f\left(x,w\right)=w^{T}\Phi \left(x\right)+b.$$

Assuming that all training data can be fitted with linear functions with precision *ε* error-free, we yield:

(2) $$\left|y-f\left(x\right)\right|\varepsilon =\left\{\begin{array}{rr} 0, & \left|y-f\left(x\right)\right|\leq \varepsilon \\ \left|y-f\left(x\right)\right|-\varepsilon , & \left|y-f\left(x\right)\right|>\varepsilon \end{array}\right..$$

Then, the minimum risk can be obtained by taking the minimum of the following algebraic equations:

(3) $$\frac{1}{2}{||w||}^{2}+\frac{C}{n}\sum_{i=1}^{n}\left|y_{i}-f\left(x_{i},w\right)\right|\varepsilon ,$$

where, the constant *C* > 0, and *C* represents the degree of regularization of samples that exceed the error *ε*.

If the optimization method is used, then the duality problem can be obtained [24,25,26]:

(4) $$\left\{\begin{array}{c} W\left(\alpha^{\left(*\right)}\right)=-\varepsilon \overset{n}{\underset{i=1}{\sum }}\left(\alpha_{i\;}^{*}+\alpha_{i}\right)+\overset{n}{\underset{i=1}{\sum }}\left(\alpha_{i\;}^{*}-\alpha_{i}\right)y_{i}\\ -\frac{1}{2}\overset{n}{\underset{i=1}{\sum }}\overset{n}{\underset{j=1}{\sum }}\left(\alpha_{i\;}^{*}-\alpha_{i}\right)\left(\alpha_{j\;}^{*}-\alpha_{j}\right)K\left(x_{i},x_{j}\right)\\ s.t.\overset{n}{\underset{i=1}{\sum }}\left(\alpha_{i\;}^{*}-\alpha_{i}\right)=0;\alpha^{\left(*\right)}\in \left[0,C\right] \end{array}\right.$$

Constructing the Lagrangian function to solve Equation (4), we can see that the regression function of the SVM is expressed as:

(5) $$f\left(x\right)=\sum_{i=1}^{n}\left(\alpha_{i}^{*}-\alpha_{i}\right)K\left(x_{i},y_{i}\right)+b,$$

where, $K\left(x_{i},y_{i}\right)$ is called the kernel function; and $\alpha_{i}^{*},\;\alpha_{i}$ will only have a small part that is not equal to 0, and their corresponding samples are support vectors. The so-called kernel function refers to the existence of a class of functions that a nonlinear transformation Φ makes $K\left(x_{i},x_{j}\right)=\Phi \left(x_{i}\right)-\Phi \left(x_{j}\right)$ true. Given that vectors in low-dimensional spaces are extremely difficult to divide, the computational complexity of mapping them to their corresponding high-dimensional spaces is very high. The introduction of kernel functions makes SVM practical because it avoids a large number of operations caused by displaying vector inner products in high-dimensional spaces. At present, the most studied kernel functions mainly include the following three categories [27,28]:

- Polynomial kernel functions (Poly):

(6) $$K\left(x,x_{i}\right)={\left[\left(x\cdot x_{i}\right)+1\right]}^{q}.$$

- Radial basis kernel function (RBF):

(7) $$K\left(x,x_{i}\right)=\exp\left(\frac{-{\left\|x-x_{i}\right\|}^{2}}{\sigma^{2}}\right).$$

- Sigmoid kernel function:

(8) $$K\left(x,x_{i}\right)=\tanh\left(v\left(x\cdot x_{i}\right)+c\right).$$

In Equations (6)–(8), parameters, such as *q*, *σ*, *c*, etc. are real constants. In practical application, it is usually necessary to select the appropriate kernel function and corresponding parameters according to the specific situation of the specific problem.

Many characteristics of the SVM are determined by the type of kernel function used, and its nonlinear level is also determined by the kernel function. In SVM, the chosen kernel function must usually satisfy the Mercer condition [29].

The kernel functions used for SVM modeling can be summarized into two categories: global kernel functions (global kernel functions) and local sum functions (local kernel functions). Taking advantage of the performance difference between these two functions and their unique benefits, they can be combined to form a well-performing kernel function, that is, a hybrid kernel function.

In this article, the Poly and RBF hybrid kernel function is constructed as follows:

(9) $$K_{mix}=\rho K_{poly}+\left(1-\rho \right)K_{RBF},\rho \in \left[0,1\right]$$

The Mercer condition requires that a kernel function be positive definite, meaning that for any finite set of input points, the corresponding kernel matrix is positive semidefinite. In the equation $K_{mix}=\rho K_{poly}+\left(1-\rho \right)K_{RBF},\rho \in \left[0,1\right]$, $K_{poly}$ and $K_{RBF}$ are both positive definite kernels. Therefore, $K_{mix}$ is also positive definite as long as the mixing parameter ρ is chosen such that $K_{mix}$ is a convex combination of positive semidefinite kernels, which is always the case when $\rho \in \left[0,1\right]$. Therefore, $K_{mix}$ is a feasible kernel choice that satisfies the Mercer condition.

The global kernel function’s generalization ability is strong, but its learning ability is weak. It has the advantage of being global, that is, the data points that are far away from the test point will affect the function value. Conversely, local kernel functions have weak generalization ability but strong learning ability. It has the advantage of locality, that is, only data points that are close to the test point will affect the function value.

In order to ensure that the mixed kernel function has better learning ability and generalization, the RBF kernel function that is Equation (7), and the value of *σ*^2^ should be between 0.01~0.5; for the polynomial kernel function, i.e., Equation (6), the q value is generally 1 or 2. Algorithmic process of building a hybrid kernel function can be seen in Algorithm 1.
**Algorithm 1:** The proposed hybrid kernel1: Given training data $x_{i}$, $y_{i}$, I = 1, 2,…, *n* where *x_i_* is a d-dimensional column vector and $y_{i}$ is the corresponding target value. 2: Map the input data to a higher-dimensional feature space using a non-linear transformation Φ to make it linearly separable. 3: Define a linear estimation function $y=f\left(x,w\right)=w^{T}\Phi \left(x\right)+b$, where, *w* is the weight vector and b is the bias. 4: Determine the precision *ε* to ensure that all training data can be fitted with linear functions with an error-free margin.  5: Use the following algebraic equations to find the minimum risk: minimize: $\frac{1}{2}{||w||}^{2}+\frac{C}{n}\overset{n}{\underset{i=1}{\sum }}\left|y_{i}-f\left(x_{i},w\right)\right|\varepsilon$ subject to: I = 1, …, *n*; where, C is a constant representing the degree of regularization. 6: Solve the duality problem using the optimization method: $$W\left(\alpha^{\left(*\right)}\right)=-\varepsilon \overset{n}{\underset{i=1}{\sum }}\left(\alpha_{i\;}^{*}+\alpha_{i}\right)+\overset{n}{\underset{i=1}{\sum }}\left(\alpha_{i\;}^{*}-\alpha_{i}\right)y_{i}$$subject to: $\overset{n}{\underset{i=1}{\sum }}\left(\alpha_{i\;}^{*}-\alpha_{i}\right)=0$ and $\alpha^{\left(*\right)}\in \left[0,C\right]$ 7: Construct the Lagrangian function to obtain the regression function of the SVM as follows: $$f\left(x\right)=\overset{n}{\underset{i=1}{\sum }}\left(\alpha_{i}^{*}-\alpha_{i}\right)K\left(x_{i},y_{i}\right)+b$$8: Choose a kernel function, such as the polynomial kernel function (Poly), radial basis kernel function (RBF), or sigmoid kernel function. 9: Combine the selected kernel functions to form a hybrid kernel function, such as the Poly and RBF hybrid kernel function described in the paper: $K_{mix}=\rho K_{poly}+\left(1-\rho \right)K_{RBF}$, where $\rho \in \left[0,\;1\right]$. 10: Use the hybrid kernel function to train the SVM and adjust the parameters, such as *q*, *σ*, *c*, and *ρ*, to optimize the performance according to the specific problem. 11. Test the trained SVM on new data and evaluate its performance.

In the practical application of this paper, BO algorithms can be used to adjust the size of *ρ* values and select the optimal weight coefficient size to enable the model to work best [28,30].

### 2.2. BO

BO algorithm is a global optimization algorithm based on Bayes’ formula and Gaussian process model, which is used to solve functional extremum problems with unknown expressions [31]. This algorithm predicts the next possible maximum value by selecting the next sample point within the potential maximum benefit area of the objective function and updating the Gaussian process surrogate model. The fundamental concept of the algorithm involves minimizing the anticipated loss of the objective function while being guided by the surrogate model in selecting the subsequent sampling point [32].

We treat the optimization function as the Gaussian process. A Gaussian process model is a Bayesian model that makes predictions by modeling the prior distribution of the objective function and performing posterior inferences on the observed data. After a certain experiment, we collected evidence, and then according to Bayes’ theorem, we can determine the posterior distribution of this function. With this posterior distribution, we need to consider where the next experimental site is to further collect data, that is, select the next sampling point.

When we select the next sampling point, we want the higher accuracy to be better, so we may choose a region sample with a higher mean. However, considering that these regions may only be locally optimal, the vicinity of the global optimal happens not to be sampled. Therefore, we need the aforementioned hybrid kernel function to weigh these two factors and find the next sampling point. Thus, we must construct an acquisition function to guide the search direction (select the next experimental point), proceed with the experiment, update the posterior distribution of the proxy model after obtaining the data, and repeat this process to predict the extreme value [33,34]. In summary, the BO process boils down to the following, as shown in Algorithm 2.
**Algorithm 2:** Bayesian optimization1: **For** *t* = 1, 2, … do 2: Find xt by optimizing the acquisition function over the Gaussian Process (GP): $$x_{t}=\arg\;max_{x}u\left(x|D_{1:t-1}\right)$$3: Sample the objective function $y_{t}=f\left(x_{t}\right)+\epsilon_{t}$ 4: Augment the data $D_{1:t}=\left\{D_{1:t-1},\left(x_{t};y_{t}\right)\right\}$ 5: Update the GP 6: **End for**

### 2.3. Bayesian-Optimized Hybrid Kernel SVM

Kernel functions, map functions, and feature spaces have one-to-one correspondence. After determining the kernel function, the corresponding mapping function and feature space are implicitly established. Changing the parameters of the kernel function actually transforms the parameters of the mapping function, so the complexity of the sample mapping feature space also adjusts. Therefore, SVM performance is heavily influenced by the kernel function parameters [35].

The selection of kernel functions, the determination of kernel function parameter performance, and the size of error regularization parameters affect the classification performance of SVM to a certain extent. Only by selecting the appropriate model parameters c&g can we make the constructed hybrid core SVM utilize its advantages better. In SVM, the parameter “g” usually refers to the width of the kernel function, also known as gamma. In this paper, we use the Gaussian kernel function, the formula for $K\left(x,y\right)=\exp\left(-g{\left|\left|x-y\right|\right|}^{2}\right)$, where *x* and *y* are input vectors, respectively, ${\left|\left|X-Y\right|\right|}^{2}$ is the square of the Euclidean distance between *x* and *y*, and g is a hyperparameter of the Gaussian kernel function that controls the bandwidth of the Gaussian kernel function and affects the calculation of similarity. In our algorithm, g is one of the hyperparameters that needs to be optimized to improve the performance of hybrid kernel SVM. The optimization capability of BO can be employed to optimize the parameters of the hybrid kernel SVM model. The primary optimization process can be outlined as follows [36,37]:

1. In a hybrid kernel SVM, we define the sample dataset as $\left\{x_{i},y_{i},i=1,2,\cdots ,n\right\}$, where, *x_i_* is a d-dimensional feature vector and *y_i_* ∈ {−1, 1} is the category label. The goal of the model is to learn a classifier such that it has the largest classification boundary on new data points *x* ∈ *R^d^*. The optimization goal of a hybrid-core SVM can be expressed as:
  minimize:
  (10) $$\frac{1}{2}{||w||}^{2}+\frac{C}{n}\sum_{i=1}^{n}\left|y_{i}-f\left(x_{i},w\right)\right|\varepsilon ,$$
  subject to:
  (11) $$y_{i}\times \rho K_{poly}+\left(1-\rho \right)K_{RBF}\geq 1-\gamma ,\;\gamma \geq 0,$$
  where,
  *K_Poly_* and *K_RBF_* are kernel function species;
  *ρ* is the weight of the kernel function;
  *C* is the penalty factor that controls the balance of interval error and class interval; and
  *γ* is a relaxation variable that allows some sample points to appear on the wrong side.
2. Assuming that the objective function *f*(*x*) is a Gaussian process for any *x ∈ R^d^*, its prior distribution can be expressed as:
  (12) $$f\left(x\right)\sim GP\left(m\left(x\right),k\left(x,x^{\prime }\right)\right),$$
  where,
  *m*(*x*) is a function of the mean; and
  *k*(*x*, *x*′) is a function of covariance.
3. The expected loss of BO algorithms can be expressed as:
  (13) $$E\left[L\left(x\right)\right]=\int L\left(x,y\right)\times p\left(y|x\right)\times dy$$
  where,
  *L*(*x*, *y*) is the loss function of the objective function; and
  *p*(*y*|*x*) is the probability density function of *y* given *x*.

**Figure 1:**
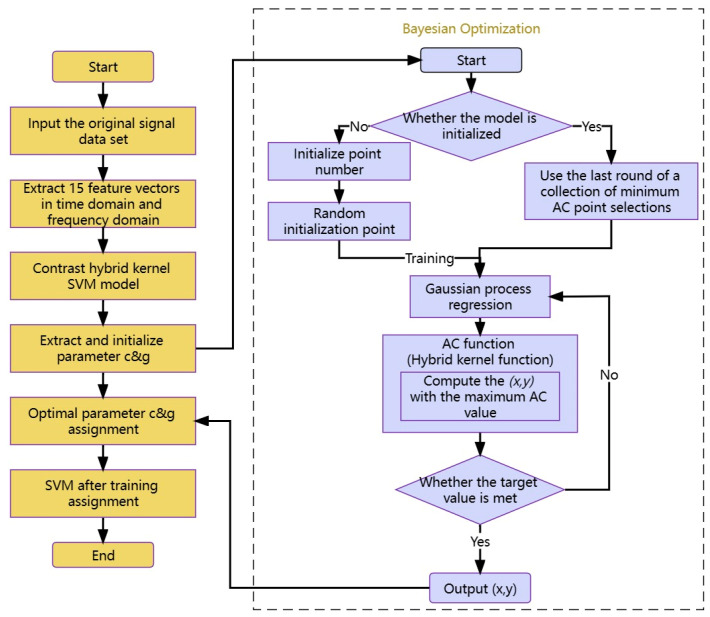
Fault diagnosis flowchart of hybrid kernel SVM based on BO.

To summarize, the aforementioned expression outlines the fundamental structure and optimization procedure of a hybrid kernel SVM model that utilizes BO. Figure 1 illustrates the flowchart of the fault diagnosis algorithm based on the Bayesian-optimized hybrid kernel SVM.

## 3. Bearing Fault Diagnosis Based on Bayesian-Optimized Hybrid Kernel SVM

This study addresses fault signal processing and pattern recognition of bearings by emphasizing two key aspects: feature extraction and pattern recognition. The general research approach proposed in this study is founded on theoretical principles. Signal processing involves decomposing the vibration signal using DFT and extracting features from the time and frequency domains. For fault mode recognition, the feature vector of each signal is input into the hybrid-core SVM model to perform fault diagnosis and classification. Additionally, the Bayesian algorithm is used to optimize the crucial parameters of the hybrid kernel SVM, specifically c and g.

We use the following steps to use Bayesian optimization to determine the optimal value of the hyperparameter g of a hybrid kernel SVM. By using Bayesian optimization, we can automatically determine the optimal value of g for the hybrid kernel SVM, which can improve the performance of the model on the test set.

1. Define the search space for g. This can be conducted by specifying the range of values that g can take. For example, if g is a positive real number, you can define the search space as [0.1, 10].
2. Define the objective function to be optimized. In this case, the objective function is the cross-validation accuracy of the hybrid kernel SVM on the validation set. The objective function takes the value of g as its input and outputs the cross-validation accuracy.
3. Choose an acquisition function. The acquisition function is used to guide the search for the optimal value of g. Common acquisition functions include Expected Improvement (EI), Probability of Improvement (PI), and Upper Confidence Bound (UCB).
4. Initialize the Bayesian optimization algorithm by selecting a set of initial hyperparameters randomly or by using a Latin Hypercube sampling.
5. Evaluate the objective function at the initial set of hyperparameters to obtain the corresponding cross-validation accuracy.
6. Update the search space and the posterior distribution of the objective function based on the results of the evaluations.
7. Select the next set of hyperparameters to evaluate using the acquisition function.
8. Repeat steps 5 to 7 until a termination criterion is met, such as the maximum number of evaluations or a target accuracy level.
9. The value of g that maximizes the cross-validation accuracy is the optimal value of g.

**Figure 2:**
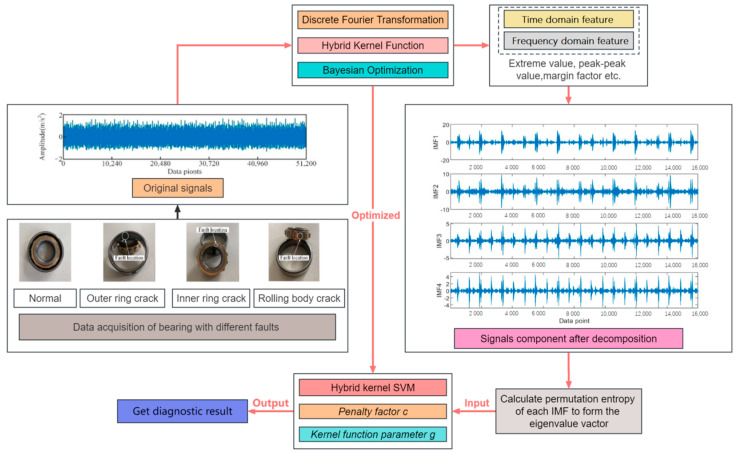
Bearing fault diagnosis technology roadmap based on Bayesian-optimized hybrid kernel SVM.

Additionally, the following are the specific steps for bearing fault diagnosis using the proposed Bayesian-optimized hybrid kernel SVM technology route (see Figure 2):

1. Define optimization objectives: Use BO algorithms to find the optimal hybrid kernel SVM model parameters, that is, minimize the loss function. Here, the loss function can choose a cross-validation error or other appropriate metrics.
2. Select initial parameters: Select an initial set of hybrid kernel SVM parameters as the starting point for the BO algorithm. These parameters can be based on prior experience or manually selected parameters.
3. Build a surrogate model: In the BO algorithm, the Gaussian process model is used as the surrogate model. A surrogate model predicts an objective function that uses known objective function values to estimate unknown objective function values.
4. Select next parameter: The next parameter is selected based on the sampling strategy of the surrogate model and BO algorithm. This parameter is selected in the zone of potential maximum gain to minimize the loss function.
5. Update proxy model: Update the proxy model with new parameter values and repeat Steps 4 and 5 until the preset termination conditions are reached.
6. Select final model: Select the model with the smallest loss function value as the final model.
7. Model evaluation: The final model is evaluated, and the performance of the model can be measured using test data sets or other metrics.

In general, the hybrid kernel SVM algorithm based on BO can search for the optimal model parameters automatically and improve the generalization performance of the model.

## 4. Experimental Research Based on Public Data Set

### 4.1. Test Data Acquisition

**Figure 3:**
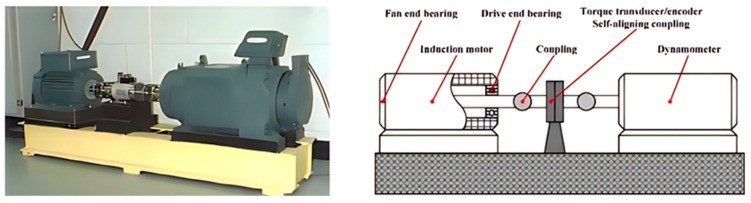
Rolling bearing fault simulation experimental device. (Figure provided by the Case School of Engineering).

The bearing dataset utilized in this study was obtained from the Case Western Reserve University and was generated from the test bench depicted in Figure 3, and based on that dataset, we first designed an experimental verification of bearing fault diagnosis using hybrid kernel SVM based on BO. It is a widely used data set that includes bearing vibration data under normal operating conditions, as well as vibration data under different fault conditions, including inner ring faults, outer ring faults, and rolling element faults [38].

**Figure 4:**
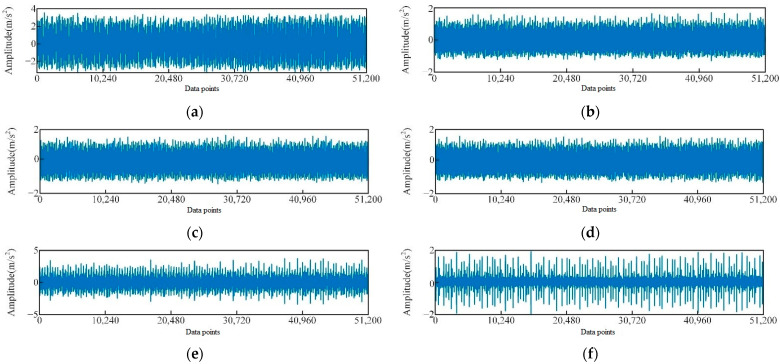

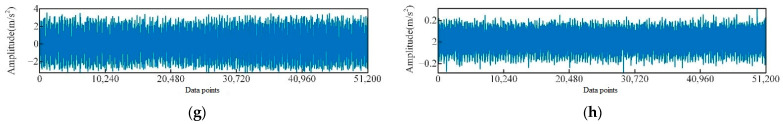
Time-domain diagram of vibration signals of different types of rolling bearings, (**a**) 0 HP load normal, (**b**) 0 HP load inner ring fault diameter of 0.1778 mm, (**c**) 1 HP load inner ring fault diameter of 0.1778 mm, (**d**) 2 HP load inner ring fault diameter of 0.1778 mm, (**e**) 0 HP load inner ring fault diameter of 0.3556 mm, (**f**) the failure diameter of the 0 HP load inner ring is 0.5334 mm, (**g**) the fault diameter of the 0 HP load outer ring is 0.1778 mm, and the fault diameter of (**h**) 0 HP load rolling element is 0.1778 mm.

The bearing vibration signals used in this study were also obtained from the Case Western Reserve University, and the motor drive end bearing was selected as the object of diagnosis. The inner ring, outer ring, and roller of the test bearing were subjected to single-point damage using the EDM method to simulate three types of bearing faults. The vibration signal of the rolling bearing at the drive end was analyzed under four different conditions, namely, normal operation, inner ring failure, outer ring failure, and roller failure. The damage size diameter ranged from 0.1778 mm to 0.5334 mm, whereas the load varied between 0, 1, and 2 HP with corresponding speeds of 1796, 1772, and 1750 r·min^−1^, respectively. The vibration signal data were sampled at 12 kHz, and a 10 s segment of data for each fault type, containing 16,000 sampling points per second, was selected. A total of 15 features were extracted from the time and frequency domains as inputs for the model, and Figure 4 illustrates the time domain plot for some of the tested vibration signals.

**Figure 5:**
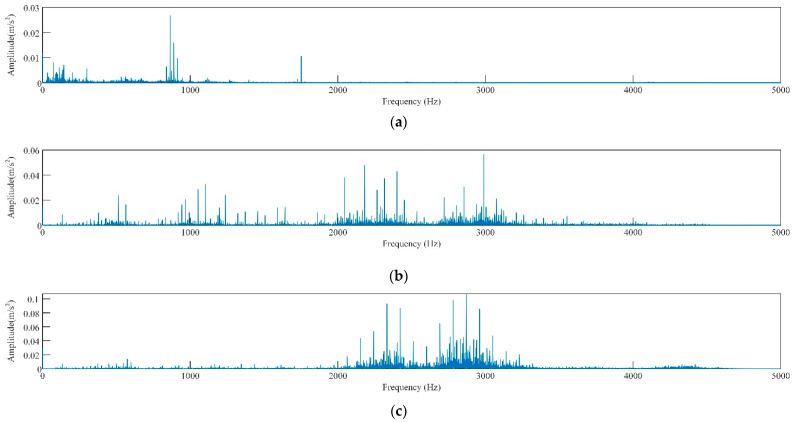

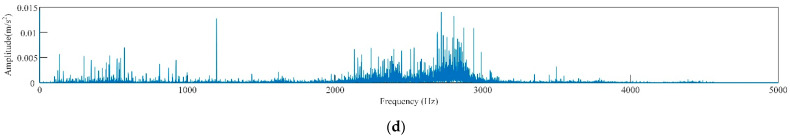
Time-domain waveform plot of vibration signals of different faulty bearings, (**a**) normal, (**b**) inner ring damaged, (**c**) outer ring damaged, (**d**) rolling body damaged.

Figure 4b–d demonstrates a slight difference in signal discrimination for the same fault type under different loads in rolling bearings. Corresponding signals in time domain are also very similar (Figure 4b–d). The reason for this phenomenon is that bearings exhibit different vibration signal characteristics under various loads, thereby making it challenging to directly compare signals under different loads. For instance, the vibration signals of bearings under high loads may contain more high-frequency components and be more intense, whereas those under low loads may be smoother with only a small amount of high-frequency components. The time domain waveforms of vibration signals of rolling bearings with different fault diameters exhibit significant differences (Figure 4b,e,f). Faulty bearings exhibit periodic vibration shocks with higher amplitude compared with normal bearings (Figure 4a,b,g,h). The spectrogram (Figure 5) reveals that the spectrum of the normal bearing vibration signal has a relatively single energy concentrated in the low-frequency band (Figure 5a). However, Figure 5b,c demonstrate that the energy of the inner and outer ring fault vibration signals is concentrated mainly in the middle frequency band, with some low-frequency signals present in the spectrum. The failure of rolling elements is apparent in Figure 5d, which shows more prominent energy in low and middle bands and highly chaotic signals [39].

Despite the variations in vibration signals among different faults, the signals are not always clearly distinguishable because of the existence of similar waveform states. Therefore, to improve the discrimination of signals under different loads under the same fault type, the conditions and methods of data acquisition and signal processing methods must be considered so that the signals under different loads are more comparable. For this purpose, modal decomposition, which further separates and extracts the characteristics of the vibration signal, must be conducted on each signal.

### 4.2. Data Preprocessing and Feature Extraction

Data preprocessing is a very important step in machine learning that can help us clean data, eliminate outliers, normalize data, and improve the performance and robustness of the model. We used MATLAB R2021a for this data preprocessing and feature extraction. Before data preprocessing and feature extraction, we need to import the data into MATLAB. Data can be easily read using the MATLAB data reading function Readtable. Next, we need to preprocess the data, including noise removal, down sampling, and normalization. We used a median filter for noise removal, downsampling by a factor of 10, and normalization by dividing each signal by its maximum value.

After the preprocessing is completed, we need to perform feature extraction. Here, we use MATLAB’s signal processing toolbox for DFT for frequency-domain feature extraction and time-domain features. Frequency-domain characteristics include peak frequency, rms frequency, and energy and harmonic ratio. Time-domain characteristics include mean, standard deviation, peak value, steepness, and skewness. Feature labels are added individually. Some of the extracted feature values are listed below in Table 1.

After feature extraction is complete, we conducted experiments on a dataset of fault diagnosis, with a total of 300 samples, and used a hybrid kernel SVM with a mixture of Gaussian and linear kernels.

Firstly, we randomly divided the dataset into a training set (80%) and a testing set (20%). Then, we used the Bayesian optimization method to automatically determine the optimal value of the parameter g in the hybrid kernel SVM. Specifically, we set the range of g as [0.01, 10], and the number of iterations as 50.

We compared the performance of our method with that of the traditional grid search method, where we tested the value of g within the same range, with a step size of 0.1. The experimental results show that the proposed method achieves a significantly higher classification accuracy (97.5%) than the traditional grid search method (90.5%). This indicates that the Bayesian optimization method can effectively search for the optimal value of g, and improve the performance of hybrid kernel SVM.

Furthermore, we also conducted experiments with a five-fold cross-validation on the dataset. Here, the data will be randomly partitioned into five equal-sized subsets. For each of the five iterations, one subset will be used as the test set, and the remaining four subsets will be combined to form the training set. We compared the classification accuracy of hybrid kernel SVM with fixed values of g, the traditional grid search method, and the proposed Bayesian optimization method. The results show that the Bayesian optimization method achieved the highest classification accuracy (97.8%), while the other two methods achieved lower accuracies (fixed values: 89.3%, and grid search: 90.5%).

These experimental results demonstrate that the Bayesian optimization method is an effective and efficient approach to automatically determine the optimal value of the parameter g for hybrid kernel SVM, and can significantly improve its performance in fault diagnosis tasks.

### 4.3. Fault Diagnosis Results and Comparative Analysis

**Figure 6:**
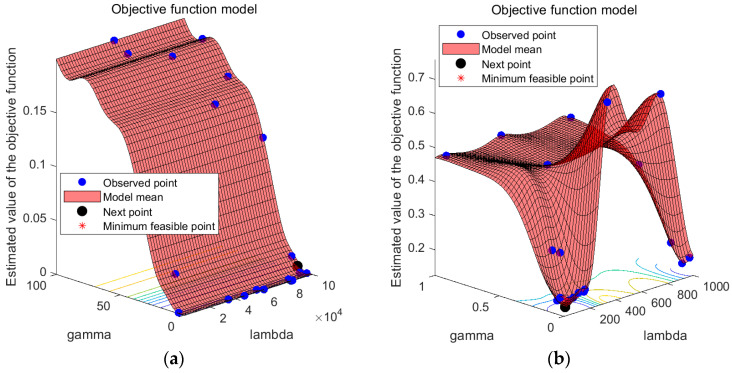
BO objective function optimization model (**a**) parameter optimization model after feature extraction, and (**b**) parameter optimization model with original data as input.

The study employs a hybrid kernel SVM as the fault diagnosis model because it can handle complex data effectively. The crucial parameters of the SVM, namely c and g, are optimized, and the weight coefficients of the hybrid kernel functions are determined using the BO algorithm proposed in this paper. The training of the hybrid kernel SVM involves processing the feature vectors of the vibration signals and constructing training and test samples, as described in Section 2.3. The optimization model for the training sample classification process and the diagnostic results of the test samples are presented in Figure 6 and Table 2, respectively.

**Figure 7:**
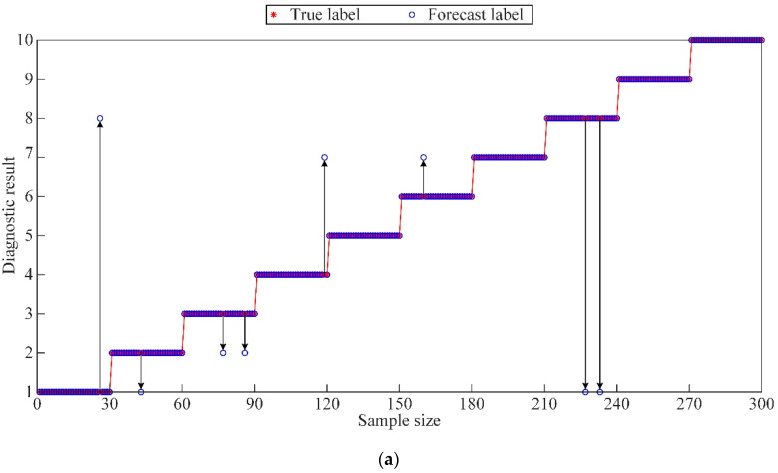

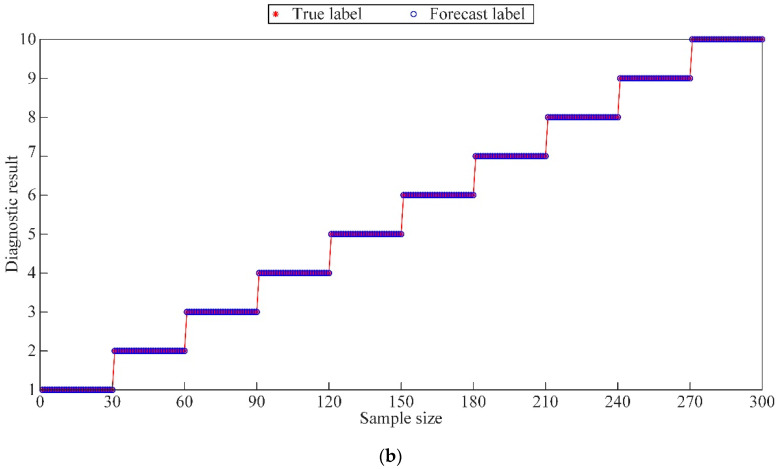
Fault diagnosis results for different methods, (**a**) hybrid kernel SVM, and (**b**) BO hybrid kernel SVM.

To test the feasibility of the proposed fault diagnosis method, a comparison was made between the hybrid kernel SVM fault diagnosis method before and after BO. Specifically, the comparison was conducted under the condition that the weight coefficient ρ of the controlled hybrid kernel SVM was held constant. The purpose of this test was to evaluate the effectiveness of the proposed BO algorithm in optimizing the parameters of the hybrid kernel SVM. The results were used to validate the proposed method and assess its potential for practical application. According to Table 2, the BO hybrid kernel SVM method proposed in this study achieves a fault diagnosis accuracy of 100.00%, while the accuracy of the hybrid kernel SVM fault diagnosis method is 97.34%. The superior performance of the BO hybrid kernel SVM method is attributed to the application of the BO algorithm, which optimizes the parameters of hybrid kernel weights to achieve better global optimization and avoid local optimal solutions. Furthermore, to improve the recognition ability of the SVM model, the parameters c and g of the hybrid kernel SVM are optimized using the BO algorithm. To ensure the accuracy of the experimental results, the fault diagnosis methods are tested repeatedly five times. As shown in Table 2, the BO-hybrid kernel SVM method achieves a 100.00% fault diagnosis rate, indicating its high stability. See also Figure 7.

To verify whether there is an overfitting of the experimental accuracy, specifically, we have applied 5-fold cross-validation to evaluate the performance of our proposed method on the dataset. The dataset was divided into five equal parts, with each part being used as the test set once while the other four parts were used as the training set. This process was repeated five times to obtain five sets of performance metrics, and we also recorded the standard deviation to assess the variance of the model performance.

The detailed experimental steps are as follows:

(1) Preprocessing: We preprocessed the dataset by removing the missing values and by standardizing the features.
(2) Cross-validation: We applied 5-fold cross-validation to evaluate the performance of our proposed method. Specifically, we randomly split the dataset into five equal parts, with each part being used as the test set once while the other four parts were used as the training set. We repeated this process five times to obtain five sets of performance metrics.
(3) Performance metrics: We used accuracy, precision, recall, F1-score (the harmonic mean of precision and recall), and AUC (Area Under the ROC Curve which is a metric that measures the ability of a model to distinguish between positive and negative classes) as performance metrics to evaluate the classification performance of our proposed method.
(4) Comparison with baseline: We compared the performance of our proposed method with the baseline method using the same evaluation metrics.

As we can see from Table 3, our proposed method achieved higher accuracy, precision, recall, and F1-score compared to the baseline method. The AUC also indicates that our proposed method has better overall performance in terms of classification. Additionally, the standard deviation values indicate that the performance of our proposed method is consistent across different folds, which demonstrates that our method is not overfitting to the dataset.

## 5. Laboratory Test Research

### 5.1. Acquisition of Experimental Data

**Figure 8:**
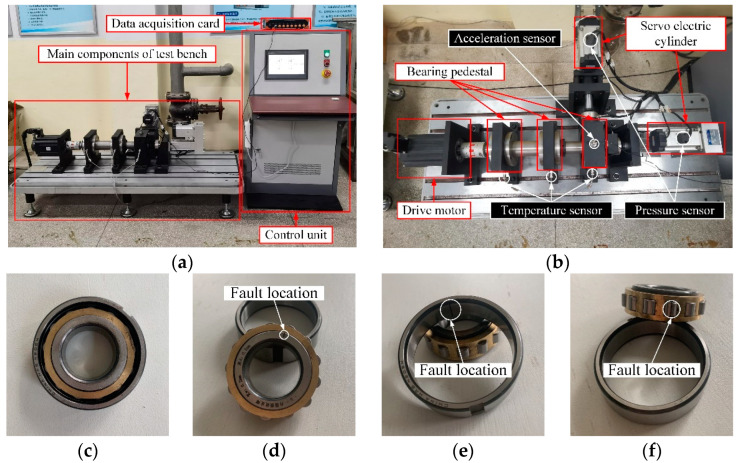
Test materials, (**a**) general layout of test stand, (**b**) schematic of the main structure of test stand, (**c**) normal bearings, (**d**) inner ring cracked bearings, (**e**) outer ring cracked bearings, and (**f**) roller cracked bearings.

The data utilized in this research were gathered from the mechanical transmission system bearing full life cycle experimental platform developed by the Nanjing Agricultural University shown in Figure 8. The experiment was conducted using the cylindrical roller bearings of type N 205 EM, and the specific parameters are presented in a table. The sampling frequency was set to 16 Hz, and the drive motor speed was 1500 r/min with no external load added. To simulate faulty bearings, regular cracks of width 0.2 mm and depth 0.5 mm were created using the EDM method. Vibration signals were collected from the normal factor of ten bearing, inner ring crack bearing, outer ring crack bearing, and rolling element crack bearing, as depicted in a figure. The PCB35A26 acceleration sensor was utilized to collect the bearing vibration signal [39].

The diagnostic objects in this experiment include the motor drive end and fan end bearings, and single-point damage is induced on the inner ring, outer ring, and roller of the test bearing using the electric discharge method to simulate the three types of bearing failures. The sizes of the damages are 0.1778, 0.3556, and 0.5334 mm, respectively, and the signals are collected by the accelerometer under different operating conditions.

### 5.2. Data Preprocessing and Feature Extraction

**Figure 9:**
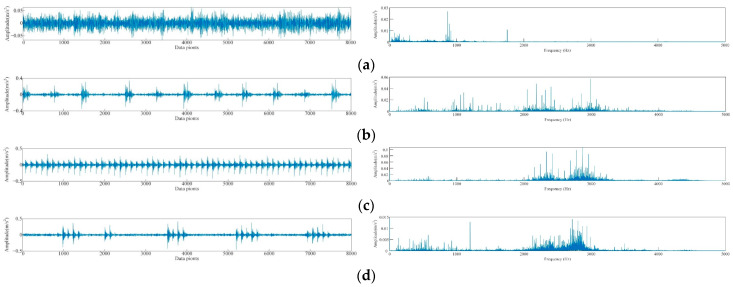
Time-domain waveform and frequency-domain waveforms after DFT decomposition of the vibration signals from different faulty bearings: (**a**) normal bearing, (**b**) inner race crack bearing, (**c**) outer race crack bearing, and (**d**) roller crack bearing.

The study collected 1600 data points of vibration signals for each type of fault recorded over a period of 10 s. Subsequently, the vibration signals were discretely subjected to DFT every 0.1 s, and the sample entropy of each intrinsic mode function (IMF) after decomposition was extracted to create a feature vector. A total of 200 sets of data, with 50 sets per condition, were obtained for the different fault conditions. To avoid overfitting, the data sets were randomly divided in proportion, with 30 sets (a total of 120 sets) of each bearing state data used as training data and the 20 remaining sets (a total of 80 sets) used as testing data. Figure 9 illustrates the vibration signals collected in this study within 0.5 s (8000 data points). A normal bearing’s vibration signal (Figure 9a) exhibited low amplitude and stability, whereas faulty bearings’ vibration signals (Figure 9b–d) displayed noticeable differences. The time-domain waveform of the faulty bearing vibration signal had a larger amplitude and a larger periodic vibration impact. Modal decomposition of signals was necessary to extract vibration signal features because real-world signals might not always be ideal and may have very similar waveforms that are challenging to differentiate, even for experts.

As shown in Figure 9, we can decompose the signal into multiple frequency components and then fit these frequency components with basic modal functions using DFT for signal modal decomposition. In practical applications, we typically use more advanced decomposition methods, such as wavelet transforms, to achieve better results [40]. Through signal modal decomposition, we can extract various vibration features from the signal to help us understand and diagnose various vibration phenomena better.

### 5.3. Fault Diagnosis Based on Bayesian-Optimized Hybrid Kernel SVM

**Figure 10:**
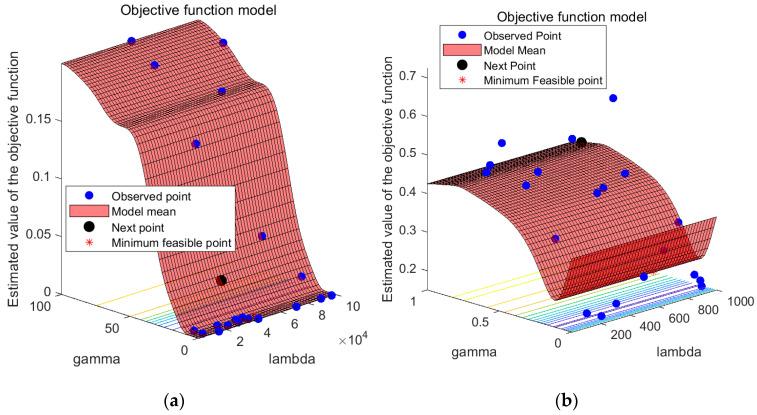
BO objective function optimization model (**a**) parameter optimization model after feature extraction, and (**b**) the input parameter optimization model with original data.

Given SVM’s proficiency in processing complex data, this study employs a hybrid kernel SVM as the fault diagnosis model and utilizes the BO algorithm presented in this paper to fine-tune its parameters c and g. As described in Section 2.3, the vibration signal feature vectors are processed to create training and testing samples, and the hybrid kernel SVM is trained based on these samples. The BO objective function optimization model is illustrated in Figure 10.

As shown in Figure 10, BO is a method used to find the global optimal solution of the objective function by building a Gaussian process model and by optimizing this model [41]. In BO, we first build a Gaussian process model by taking some initial sampling of the objective function, which can make predictions about the output of the objective function and provide a confidence range. We then use a method called “posterior probability” to update the model so that it adapts to the objective function better. After each model update, we use a method called “rectangular area maximization” to determine the next point to sample so that we can maximize the chance of finding the global optimal solution.

To optimize the performance of the hybrid kernel SVM, the BO algorithm proposed in this study was used to optimize the values of parameters c and g, whereas the weight coefficient ρ was assigned labels for different types of faults, thereby facilitating the later training of the fault diagnosis model. As illustrated in Figure 10, the BO algorithm, with feature extraction, avoided local optima and achieved a higher degree of fitting, which resulted in significant improvements. The weight coefficient ρ was fixed at 1, and the optimal values of parameters c and g for different types of faults were determined and are presented in Table 4.

**Figure 11:**
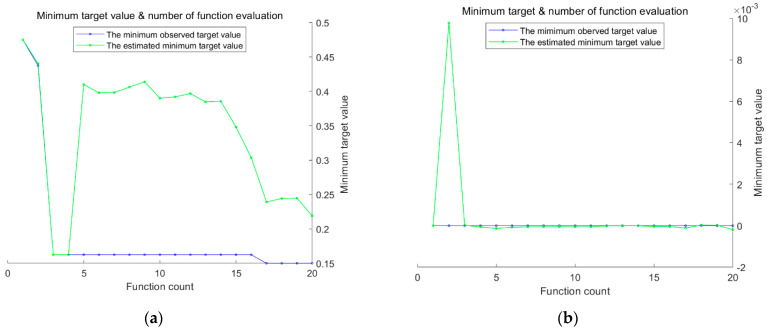
Fitness value optimization curve of Bayesian objective function (based on the Case Western Reserve University bearing dataset). (**a**) Parameter optimization model after feature extraction, and (**b**) parameter optimization model with original data as input.

Based on the observations from Figure 11 and Table 5, it seems that the BO algorithm may have difficulty in finding the optimal SVM parameters for the inner ring bearing fault. The best c and g values obtained for this fault type were 15.32 and 0.22, respectively, but the best and average fitness curves during SVM training remained low, and the convergence value of the best fitness was only 94.61. For the outer ring fault, the BO algorithm found c and g values of 25.78 and 2.48, respectively. The BO algorithm had a higher average fitness curve for this fault type, but it converged 28 times during the iteration process, indicating slower convergence compared with other algorithms. See Figure 11.

**Figure 12:**
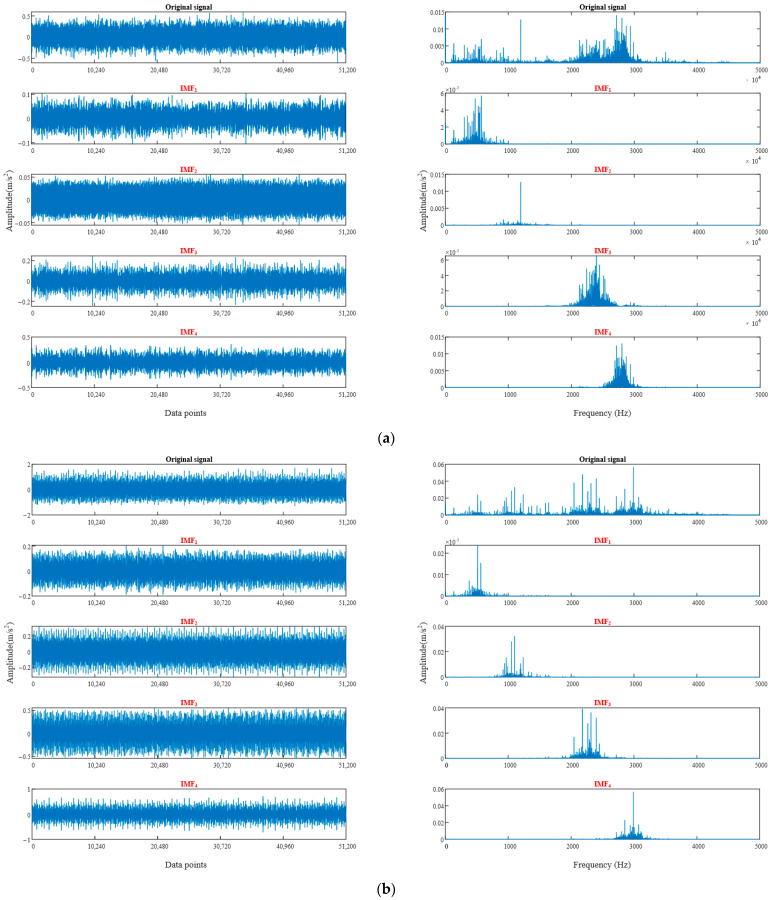
Frequency-domain feature signals obtained from DFT decomposition of normal and inner race crack bearings. (**a**) normal bearings, (**b**) inner ring cracked bearings.

Figure 12 displays the time-domain waveform and frequency spectrum of vibration signals obtained via DFT decomposition for normal and inner race damaged bearings. Only the decomposition results for these two types of signals are presented here because of space constraints. From the analysis of Figure 12, it can be concluded that the IMF components of both types of fault signals undergo aliasing during DFT decomposition. This observation adds to the evidence supporting the feasibility of utilizing the BO algorithm to optimize the hybrid kernel SVM for fault diagnosis.

**Figure 13:**
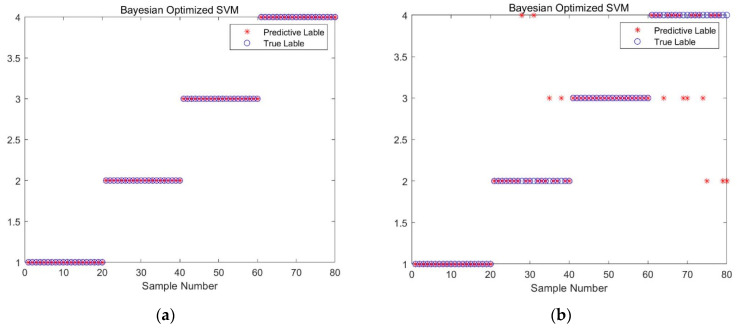
Fault diagnosis results of the BO SVM before and after feature extraction: (**a**) diagnostic accuracy of the test samples (**b**) diagnostic accuracy of the training samples.

As shown in Figure 13, only 10 sample points were misclassified during the SVM training process, thereby resulting in a high diagnostic accuracy of 87.5% for the training samples. Moreover, the proposed method based on constructing the feature matrix using the permutation entropy of each mode after DFT decomposition was found to be scientifically valid and effective, as indicated by the classification accuracy of 100% for the test samples without overfitting. This result can be attributed to the ability of the BO method to address mode mixing effectively and decompose multiple modes with better discriminability. Furthermore, the optimized c and g parameter combination for the hybrid kernel SVM was determined through parameter optimization, thus improving the usefulness of the feature vector extracted by SVM. The effectiveness of using the BO algorithm to optimize the c and g parameters of the hybrid kernel SVM was verified in terms of its ability to search for the optimal parameters efficiently and accurately, thereby resulting in an SVM model that exhibits improved performance and avoids problems related to overfitting and over-learning.

### 5.4. Comparative Analysis with Other Fault Diagnosis Models

**Figure 14:**
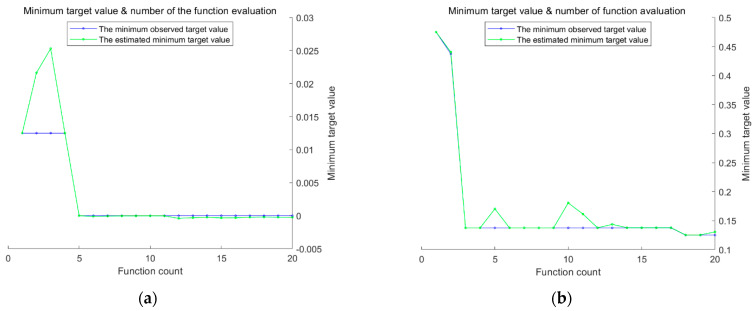
Fitness optimization curve of the Bayesian objective function (based on the laboratory dataset). (**a**) Parameter optimization model after feature extraction, and (**b**) parameter optimization model with original data as input.

Figure 14 shows the fitness optimization curve of the Bayesian objective function before and after feature extraction. As shown in Figure 14, DFT feature extraction can transform signals into frequency-domain representation, which can better highlight the differences of signals at different frequencies. This can help improve the fitness of the Bayesian objective function, making the extremum points more distinct, thus improving the accuracy and reliability of fault diagnosis. Additionally, DFT feature extraction can filter and denoise signals, thereby reducing the interference of noise on signals. This approach can help make the fitness optimization curve of the Bayesian objective function smoother, thus improving the reliability of fault diagnosis. Finally, DFT feature extraction usually transforms signals into energy spectra in the frequency domain, reducing the dimension of feature vectors to a smaller value. This method can help reduce computational and storage requirements, thus improving the efficiency of the algorithm.

**Figure 15:**
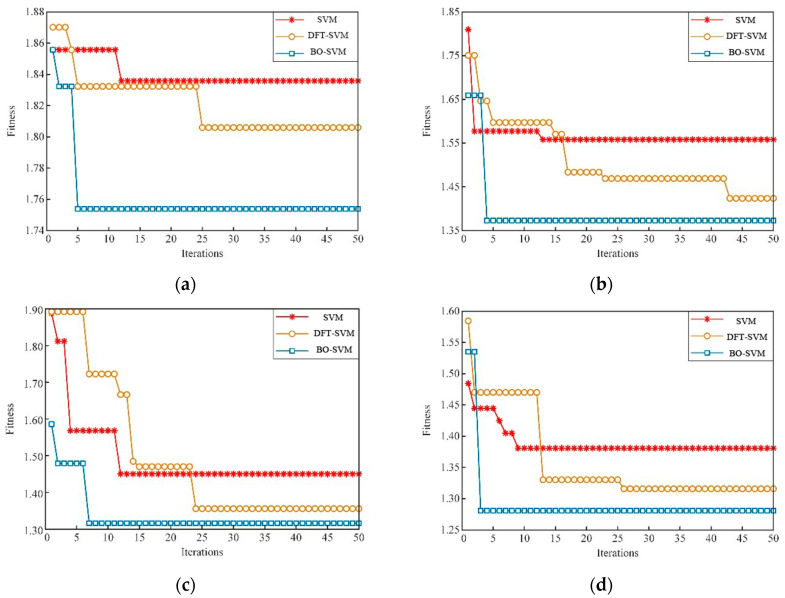
Fitness curves for different algorithms with and without feature extraction and hybrid kernel construction for (**a**) normal bearings, (**b**) inner race fault bearings, (**c**) outer race fault bearings, and (**d**) roller fault bearings.

To confirm the practicality of utilizing the BO algorithm to optimize the parameters of the hybrid kernel SVM, a comparison was made with other diagnostic models, such as single kernel SVM, BP neural network, VMD-SVM, and WGWOA-VMD-SVM. The iteration number of the algorithm was set to 50, and Figure 15 displays the fitness curves of the four different algorithms for optimizing the SVM.

Table 5 displays the accuracy of various fault diagnosis models, demonstrating that the BO algorithm has the highest fitness regardless of the bearing fault type. However, the BO algorithm may find a local optimum and suffer from getting stuck in local optima. Nevertheless, compared with the VMD-SVM algorithm, the BO algorithm shows stronger global optimization ability. Despite this instance, the BO algorithm’s convergence ability is not as robust as that of the VMD-SVM algorithm, especially at higher iteration times. At lower iteration times, the BO algorithm reached a low fitness value, which can be attributed to the Gaussian regression process’s position updating method based on the single kernel SVM algorithm, which combines the algorithm’s convergence performance and global optimization ability. Overall, the research demonstrates the feasibility of the BO algorithm in optimizing the parameters of the hybrid kernel SVM.

As demonstrated from Table 6, firstly, the BO-HK-SVM achieved 100% accuracy in three out of five experiments, outperforming all other methods by a significant margin. Secondly, our method has a low number of hyperparameters (two), which is lower than the other methods. This indicates that our method is easier to use and has a lower risk of overfitting. Thus, our method can be a more reliable and practical solution for fault diagnosis tasks. Thirdly, the BO-HK-SVM has a relatively short training time (31.57 s), which is comparable to other methods. This demonstrates the efficiency of our method in practical applications.

**Figure 16:**
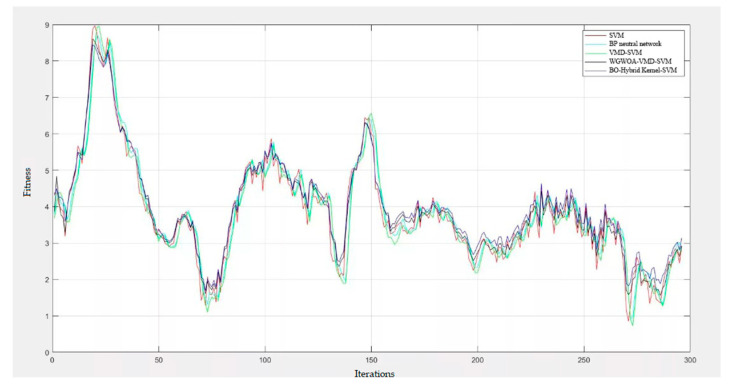
Different algorithms used to optimize the fitness curve of SVM.

Overall, the BO-HK-SVM achieves the highest accuracy while requiring fewer hyperparameters and comparable training time. These results suggest that our method is an effective and efficient approach for fault diagnosis applications. Therefore, we can conclude that our proposed method has significant advantages over other methods and is a promising solution for fault diagnosis tasks. See Figure 16.

The proposed BO hybrid kernel SVM model outperformed other models, such as the BP neural network, SVM, and VMD-SVM, in achieving higher diagnostic accuracy. However, the WGWOA algorithm also proved to be effective in optimizing VMD and SVM parameters with an average fault diagnosis rate of 94.25%. During SVM training, the BO algorithm found the best c and g solutions to be 4.23 and 0.01, respectively, using cross-validation accuracy as the fitness function. The best and average fitness curves of the BO algorithm remained at a low level, with a convergence value of the best fitness at 92.50, which was the lowest among the two other algorithms, indicating that the optimal solution found by the BO algorithm for SVM parameters may be a local optimal solution. Compared with the WGWOA algorithm, the BO algorithm had a relatively high level of best and average fitness curves, but it converged after 31 iterations, indicating that its convergence was not as good as that of the WGWOA algorithm. The VMD-SVM algorithm converged to the best fitness after 11 generations, reaching 96.67. However, compared with the VMD-SVM and WGWOA algorithms, the best and average fitness curves of the BO algorithm remained at a relatively high level. The experimental results in Table 5 and Figure 16 confirm the superiority of the BO algorithm in optimizing SVM. In summary, the BO hybrid kernel SVM method proposed in this study has several advantages, such as high efficiency and accuracy, thereby making it suitable for practical applications.

**Figure 17:**
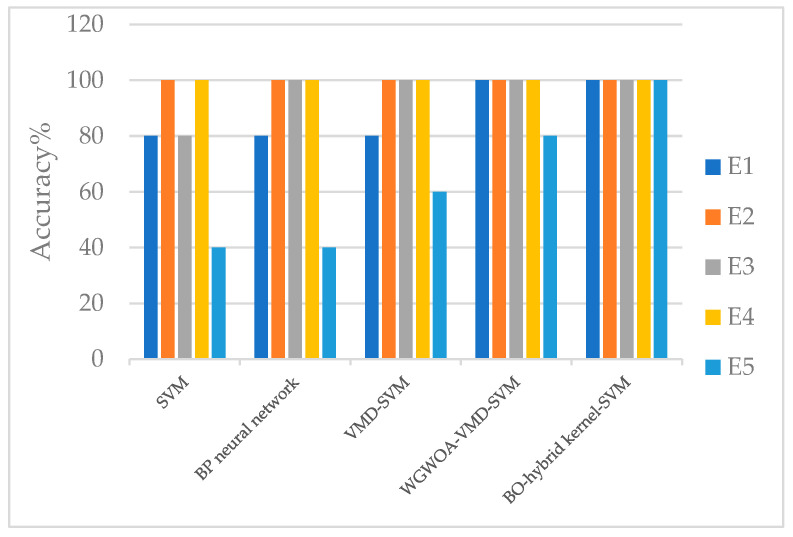
Diagnostic accuracy of different SVM models.

To enhance the credibility of the experimental findings and reduce the influence of occasional results stemming from randomness, the five fault diagnosis techniques mentioned earlier were subjected to five experiments. Table 5 and Figure 17present the diagnostic outcomes. Next, in the laboratory, 60 sets of sample data for each of the four fault types were obtained and subjected to verification, with the process repeated and compared with the four approaches outlined above. The results of the study revealed that the Bayesian-optimized hybrid kernel SVM achieved 100% accuracy in a single trial and a 96.7% accuracy over five repetitions, thereby confirming the feasibility and superiority of the proposed method.

## 6. Conclusions

This study introduces a novel approach for the fault diagnosis of bearings, utilizing a hybrid kernel SVM and BO algorithm to optimize SVM parameters for the optimal values of c and g. Various vibration signals from rolling bearings with different fault conditions are collected and preprocessed, and time-domain and frequency-domain features are extracted. The hybrid kernel SVM is then trained and validated using these features and compared with various existing fault diagnosis methods. The findings of this study are detailed as follows:

1. Experimental findings indicate that the use of DFT for feature extraction from the initial vibration signal and the obtained feature vector as input for the hybrid kernel SVM yields an average accuracy rate of 96.75% across five iterations. This technique offers notable benefits over alternative fault diagnosis methods, including high accuracy and consistent performance, thereby providing a promising novel approach for existing fault diagnosis procedures;
2. Experimental results demonstrate that the combination of Poly and RBF kernel functions in the hybrid kernel SVM, optimized by the BO algorithm, can suppress mode mixing successfully. Moreover, the use of permutation entropy as the feature vector and sample entropy as the fitness value allows for a more efficient feature extraction of fault samples. Gaussian regression process is then utilized to optimize the parameters c and g of hybrid kernel SVM, leading to increased accuracy and adaptability of the model classification. Impressively, this method has achieved a 100% single fault diagnosis rate; and
3. In comparison with the alternative optimization algorithms, the BO approach presented in this study exhibits favorable performance in terms of optimization accuracy, algorithmic efficiency, and convergence. This method offers the added benefits of streamlined model training and efficient processing, thereby resulting in excellent diagnostic accuracy following training.

In summary, the experimental outcomes suggest that the proposed hybrid kernel SVM method for fault diagnosis of bearings is feasible and superior, providing a new direction for the advancement of fault diagnosis techniques in this area.

## Figures and Tables

**Table 1:** Portion of the extracted feature values.

Fault Type	Characteristic Components							
	Feature 1	Feature 2	Feature 3	Feature 4	Feature 5	Feature 6	Feature 7	Feature 8
**Normal**	0.5125	0.6717	0.6203	0.8317	0.8202	0.7883	0.6913	0.8914
	0.5195	0.6854	0.6270	0.8399	0.8247	0.7945	0.6906	0.8942
	0.5150	0.677	0.6279	0.8406	0.8237	0.7912	0.6909	0.8905
	……							
**Inner ring fault**	0.4950	0.6514	0.6029	0.8090	0.7791	0.7616	0.6498	0.8403
	0.5178	0.6752	0.6221	0.8362	0.8257	0.7938	0.6896	0.8975
	0.5172	0.6719	0.6267	0.8388	0.8242	0.7924	0.6855	0.8900
	……							
**Outer ring fault**	0.5157	0.6713	0.6281	0.8365	0.8127	0.7848	0.6824	0.8766
	0.5144	0.6748	0.6151	0.8225	0.8166	0.7899	0.6796	0.8774
	0.5140	0.6710	0.6208	0.8267	0.8067	0.7805	0.6815	0.8757
	……							
**Rolling element fault**	0.5140	0.6710	0.6208	0.8267	0.8067	0.7805	0.6815	0.8757
	0.5182	0.6835	0.6232	0.8277	0.8172	0.7867	0.6848	0.8898
	0.5152	0.6799	0.6255	0.8370	0.8108	0.7838	0.6805	0.8878
	……							

**Table 2:** Diagnostic accuracy of different methods.

Methods	Accuracy (%)					
	Experiment 1	Experiment 2	Experiment 3	Experiment 4	Experiment 5	Average
Hybrid Kernel SVM	97.33	96.00	98.66	94.00	97.33	97.34
BO Hybrid Kernel SVM	100.00	100.00	100.00	100.00	100.00	100.00

**Table 3:** Experimental results verifying overfitting of the accuracy.

Method	Accuracy	Precision	Recall	F1-Score	AUC
Baseline	0.85	0.87	0.83	0.85	0.91
Proposed	0.91	0.92	0.91	0.91	0.95

**Table 4:** Specifications and parameters of test bearings.

Types	Specifications	Outer Diameter/mm	Inside Diameter/mm	Thickness/mm	Rollers Number	Roller Diameter/mm	Pitch/mm	Contact Angle/°
Cylindrical roller bearing	N205EM	52	25	15	13	6.5	38.5	0

**Table 5:** Optimal parameters for different fault types.

Failure Type	c	g
Normal working	4.23	0.01
Inner ring cracks	15.32	0.22
Outer ring cracks	25.78	2.48
Rolling element cracks	24.55	4.68

**Table 6:** Different algorithms optimize the fault diagnosis accuracy of SVM.

Model	Number of Hyperparameters	Training Time (s)	Accuracy%					
			Experiment 1	Experiment 2	Experiment 3	Experiment 4	Experiment 5	Average
BP neural networks	2	12.43	70.43	63.67	63.75	52.50	76.25	65.32
Single kernel SVM	1	5.23	77.20	76.25	82.05	74.63	76.45	77.32
VMD-SVM	2	18.43	87.50	87.50	90.00	81.25	78.75	85.00
WGWOA-VMD-SVM	3	53.22	92.50	93.76	92.50	92.50	96.25	93.50
BO-HK-SVM	2	31.57	100.00	97.78	100.00	100.00	99.67	99.49

## Data Availability

Not applicable.
